# Draft Genome Assemblies of 4 Lactobacillus jensenii and 3 Lactobacillus mulieris Strains from the Urinary Tract

**DOI:** 10.1128/mra.00032-22

**Published:** 2022-04-07

**Authors:** Adriana Ene, Catherine Putonti

**Affiliations:** a Bioinformatics Program, Loyola University Chicago, Chicago, Illinois, USA; b Department of Biology, Loyola University Chicago, Chicago, Illinois, USA; c Department of Microbiology and Immunology, Loyola University Chicago, Stritch School of Medicine, Maywood, Illinois, USA; Indiana University, Bloomington

## Abstract

Lactobacilli are dominant members of the healthy female bladder microbiota. Here, we report the draft genome sequences of 4 Lactobacillus jensenii and 3 *Lactobacillus mulieris* strains isolated from catheterized urine samples.

## ANNOUNCEMENT

*Lactobacillus* bacteria are key members of the healthy urinary and vaginal microbiota (1, 2). One species, Lactobacillus jensenii, is generally considered beneficial to these communities and is capable of inhibiting or killing uropathogenic Escherichia coli (3). However, L. jensenii is isolated frequently from the bladders of females with urge urinary incontinence (UUI) (4). Recently, a new *Lactobacillus* species of the female urogenital tract was characterized—Lactobacillus mulieris (5). L. jensenii and *L. mulieris* are closely related taxa, and their genomes can best be distinguished by average nucleotide identity (ANI) (5). Using ANI, publicly available genomes of L. jensenii were reassigned as *L. mulieris* (6), and additional *L. mulieris* strains were sequenced (7, 8). In an effort to better characterize the genetic content of L. jensenii and *L. mulieris*, we have sequenced 4 urinary isolates of L. jensenii and 3 urinary isolates of *L. mulieris*.

Catheterized urine samples, collected from females as part of prior institutional review board (IRB)-approved studies (IRB approvals LUC206469, LUC207102, and LUC204195 from Loyola University Chicago and 17077AW from University of California San Diego) (9–13), were cultured using the enhanced quantitative urine culture (EQUC) method (13) and stored at −80°C. Seven strains identified as L. jensenii by matrix-assisted laser desorption/ionization-time of flight (MALDI-TOF) mass spectrometry (as previously described [9]) were selected for whole-genome sequencing. Freezer stocks were first streaked onto Columbia colistin and nalidixic acid (CNA) agar with 5% sheep blood plates (BD 221353) and incubated at 35°C in 5% CO_2_ for 48 h. A single colony was then selected and grown in MRS + 1% Tween liquid medium at 35°C in 5% CO_2_ for 48 h. The Qiagen blood and tissue kit was used for DNA extraction, following the manufacturer’s protocol with the additional lysis treatment prior to extraction. This lysis treatment includes a suspension of the cell pellet in lysis buffer (see details in reference 7) and incubation at 35°C for 30 min. The DNA was quantified using a Qubit fluorometer and sent to the Microbial Genomic Sequencing Center (Pittsburgh, PA) for library preparation (using the Illumina DNA prep kit and Integrated DNA Technologies [IDT] 10-bp unique dual index [UDIs]) and sequencing on the Illumina NextSeq 2000 platform (paired-end, 150-bp reads); demultiplexing, quality control, and adapter trimming were performed using bcl-convert (v. 3.9.3; https://support-docs.illumina.com/SW/BCL_Convert/Content/SW/FrontPages/BCL_Convert.htm). Raw reads were first trimmed for quality using bbduk (v. 38.92) (https://sourceforge.net/projects/bbmap/) with the following parameters: ftl = 15, ftr = 135, minlength = 30, qtrim=rl, maq = 20, maxns = 0, statscolumns = 5, and trimq = 20. Filtered reads were assembled via SPAdes v. 3.15.2 using the “only-assembler” option for k = 55, 77, 99, and 127 (14). Genome annotations were performed using the NCBI Prokaryotic Genome Annotation Pipeline (PGAP) v. 5.3 (15).

**FIG 1:**
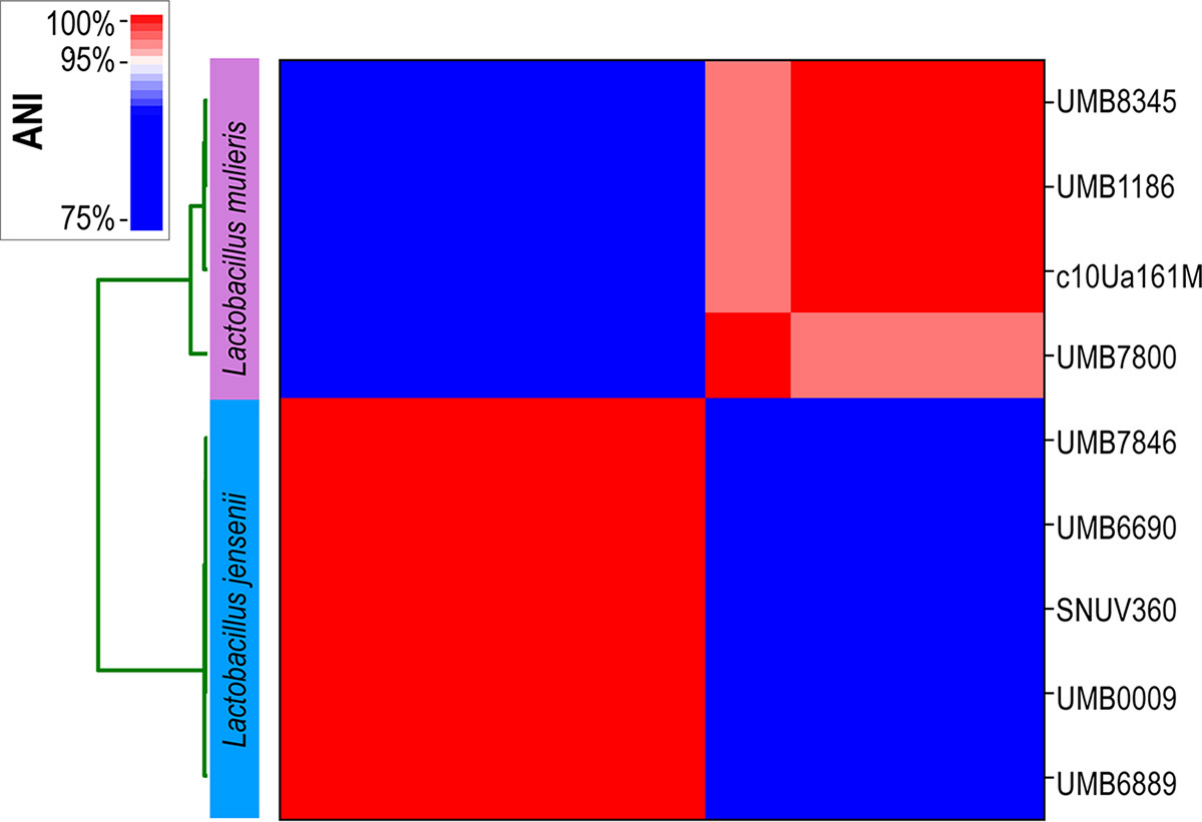
ANI analysis of genomes from 7 urinary isolates, L. jensenii SNUV360 (NZ_CP0188091), and *L. mulieris* c10Ua161M (GCA_007095465.1).

The seven genomes were compared to representative strains of the two species, namely, L. jensenii SNUV360 (NZ_CP018809.1) and *L. mulieris* c10Ua161M (GCA_007095465.1), using pyANI (v. 0.2) (16). Based upon this ANI analysis (Fig. 1), we can assign their taxonomy, as follows: 4 strains of L. jensenii and 3 strains of *L. mulieris*. The draft genome assemblies of these seven bladder lactobacilli genomes vary in size from 1,487,531 bp (L. jensenii UMB0009) to 1,803,482 bp (L. jensenii UMB7846), with an average GC content of 34.20%. Full assembly statistics are listed in Table 1. Subsequent sequencing of these two species will provide insight into their role in the urinary microbiota.

**TABLE 1:** L. jensenii and *L. mulieris* draft genome statistics

Strain name	SRA accession no.	No. of pairs of raw reads	Genome accession no.	Coverage (×)	No. of contigs	Length (bp)	*N*_50_ (bp)	GC content (%)	Symptom status^*a*^
L. jensenii UMB0009	SRR17382894	1,490,462	JAKEYG000000000	288.37	56	1,487,531	49,572	34.26	OAB−/UTI−
L. jensenii UMB6889	SRR17382891	1,500,599	JAKEYJ000000000	262.94	63	1,662,558	49,065	34.17	OAB−/UTI−
L. jensenii UMB6690	SRR17382892	1,388,309	JAKEYI000000000	233.47	55	1,741,648	56,078	34.44	rUTI
L. jensenii UMB7846	SRR17382889	1,537,023	JAKEYL000000000	250.11	45	1,803,482	73,859	34.29	OAB+/UTI−
*L. mulieris* UMB1186	SRR17382893	101,961.5	JAKEYH000000000	257.99	78	1,696,445	43,865	34.16	UTI+
*L. mulieris* UMB7800	SRR17382890	1,400,104	JAKEYK000000000	237.14	54	1,694,839	56,825	34.13	rUTI
*L. mulieris* UMB8354	SRR17382888	595,635	JAKEYM000000000	108.19	63	1,604,117	71,306	33.94	OAB+/UTI−

a Participant symptom status abbreviations are as follows: OAB, overactive bladder symptoms; UTI, urinary tract infection; and rUTI, recurrent UTI.

### Data availability.

For all seven strains, the raw reads and draft assemblies have been deposited in GenBank. Table 1 lists the SRA accession numbers and genome assembly accession numbers.
